# Peer victimization and non-suicidal self-injury among high school students: the mediating role of social anxiety, mobile phone addiction, and sex differences

**DOI:** 10.1186/s12888-024-05495-8

**Published:** 2024-01-04

**Authors:** Qianmei Long, Bin Huang, Yiyu Tang, Junlin Wu, Jia Yu, Junlin Qiu, Yanqing Huang, Guoping Huang

**Affiliations:** 1 Department of Psychiatry, North Sichuan Medical College, 637000 Nanchong, China; 2 The Third Hospital of Mianyang, Sichuan Mental Health Center, 621000 Mianyang, China; 3 The Third Hospital of Panzhihua, Panzhihua Mental Health Center, 617061 Panzhihua, China

**Keywords:** Non-suicidal self-injury, Peer victimization, Social anxiety, Mobile phone addiction

## Abstract

**Background:**

Peer victimization (PV) is one of the major causes of non-suicidal self-injury. Non-suicidal self-injury (NSSI), peer victimization, social anxiety, and mobile phone addiction are significantly related; however, the interaction mechanism and effect of sex differences remain to be determined.

**Objective:**

Herein, we investigated the relationship between peer victimization and NSSI among Chinese high school students. We also explored the chain mediating roles of social anxiety and mobile phone addiction and the regulatory role of sex. The findings of this study provide insights for theoretical interventions based on internal mechanisms.

**Method:**

A self-reported survey of 14,666 high school students from Sichuan County was conducted using a peer victimization scale, NSSI scale, social anxiety scale, and mobile phone addiction scale. A self-administered questionnaire was used to capture sociodemographic information.

**Results:**

Peer victimization, social anxiety, and mobile phone addiction were positively correlated with NSSI. Peer victimization had significant direct predictive effects on NSSI (95% *CI*: 0.341, 0.385) and significant indirect predictive effects on NSSI through social anxiety (95% *CI*: 0.008, 0.019) or mobile phone addiction (95% *CI*: 0.036, 0.053). Peer victimization had significant indirect predictive effects on NSSI through social anxiety as well as mobile phone addiction (95% *CI*: 0.009, 0.014). The first stage (predicting the effect of peer victimization on NSSI) and the third stage (predicting the effect of mobile phone addiction on NSSI) were both moderated by sex.

**Conclusions:**

Peer victimization could directly predict NSSI and indirectly predict NSSI through social anxiety and mobile phone addiction. Thus, social anxiety and mobile phone addiction exhibited chain mediating effects between peer victimization and NSSI in high school students; moreover, sex might be involved in the regulation of the mediation process.

## Introduction

### Non-suicidal self-injury

Non-suicidal self-injury (NSSI) is the deliberate self-destruction of bodily tissue for purposes that are not socially sanctioned, without suicidal intent [1]. A survey encompassing 11 European countries showed that the prevalence of NSSI was 17.1–38.6% and that the most common form of self-injury was self-cutting [2]. A recent retrospective analysis showed that even in developing countries, the prevalence of NSSI was 11.5–33.8%, showing a continuously increasing trend, which is almost similar to that in developed countries [3]. According to Victor et al. [4], NSSI is more common in adolescence and early adulthood. This may be related to the gradual maturation of the prefrontal cortex area responsible for functions such as emotional regulation during adolescence; thus, adolescents are more susceptible to changes in the environment [5, 6]. NSSI may predict future suicide attempts and increase suicide risk [6–8]. It is also associated with mental health problems and substance use disorders in adulthood [9–11] and causes harms such as deteriorated physical health and decreased life expectancy [12]. A prospective cohort study suggested that NSSI in early adolescence indicates the emergence of a new psychiatric disorder [13]. Because of the widespread and harmful nature of NSSI and its specific influence on adolescents, NSSI behavior among adolescents has become a key issue to be addressed.

### Peer victimization and NSSI

Peer victimization is defined as the experience of aggression, bullying, and social exclusion [14]. It is different from traditional bullying because in bullying, there is more emphasis on the imbalance of power [15]. Some researchers consider bullying to be a subtype of peer victimization [14]. Adolescence is a critical period during which peer victimization develops and forms roots in the mind of an adolescent [16]. The incidence of different forms of peer abuse among European adolescents is between 9.4% and 33.0% [17]. Peer victimization is typically considered a negative peer relationship [18]. Peer victimization is an important risk factor for NSSI, that is, people who experience peer victimization may be more likely to develop NSSI behaviors [19–21]. A cross-sectional survey of 2284 rural primary and secondary school students in China showed that students who had experienced peer victimization were more likely to engage in NSSI behaviors [22]. A meta-analysis of the relationship between peer victimization and NSSI showed a significant positive association between peer victimization and NSSI [20]. This may be understood in terms of the interpersonal reinforcement model of self-injury. In this case, the presence of distal risk factors that lead to problems such as interpersonal communication increases the risk of NSSI, and victims may seek help through NSSI behaviors; however, they may also avoid undesired social situations through NSSI behaviors [23]. As an important risk factor for NSSI, the internal mechanisms between peer victimization and self-injury should be urgently explored, and groups prone to these behaviors should be identified.

### Mediating role of social anxiety

Social anxiety comprises emotions such as fear, uneasiness, and distress that one experiences in interpersonal situations, it can cause individuals to adopt avoidance behaviors, and they may be hesitant to participate in similar social situations in the future [24]. The level of social anxiety among Chinese adolescents increased significantly each year [25]. Some individuals with social anxiety exhibited highly impulsive and high-risk behaviors such as self-injury, suicide, and substance abuse [26]. Some people with NSSI exhibit specific behaviors for avoiding interpersonal interactions [27, 28]; 8% of borderline and avoidant personalities engage in NSSI behaviors for avoiding social interactions [29]. Peer victimization exhibits a close relationship with social anxiety [30, 31]. According to the social self-efficacy theory [32], peer victimization can significantly reduce adolescents’ social self-efficacy and lead to negative self-evaluations. Moreover, individuals with low social self-efficacy often develop social anxiety because they cannot cope with sudden changes in social situations. A meta-analysis showed a significant positive association between peer victimization and social anxiety, and it led to persistent or aggravated social anxiety [31]. In this study, we propose that adolescent NSSI behavior may be associated with social anxiety, and peer victimization predicts social anxiety levels; therefore, social anxiety may play a mediating role between peer aggression and NSSI.

### Chain mediating role of social anxiety and mobile phone addiction

Individuals with social anxiety tend to exhibit negative cognitive patterns [33], and they tend to be negativize pessimistic about the social cues expressed by others. In online interactions such as on mobile phones and web platforms, socially anxious individuals are involved in a non-face-to-face form of social interaction, which, considering the aforementioned social self-efficacy theory, avoids the timely feedback associated with traditional face-to-face interactions. Social anxiety can predict mobile phone addiction levels [34, 35] Although mobile phone addiction has not yet been included in the DSM-V(American Handbook of Mental Disorders, Fifth Edition) and there is no consolidated definition, it is a prevalent health problem, mainly manifested by excessive use; it can disrupt social activities and when mobile phone use is prohibited, it can lead to mood swings [36]. A survey conducted by Lopez-Fernandez et al. [37] showed that the prevalence of mobile phone addiction among UK teenagers is 10%. A cross-sectional survey also demonstrated that peer victimization and mobile phone addiction are related [38]. Another longitudinal study showed that levels of peer victimization predicted levels of mobile phone addiction after six months and one year [39]. In contrast, unmonitored/unchecked mobile phone usage can cause headaches, insomnia, anxiety, depression, self-injury, and so on [40, 41]. A cross-sectional survey of 2,719 adolescents in China showed that those with higher mobile phone addiction scores were more likely to experience severe, repeated NSSI [42]. In summary, peer victimization may lead to social anxiety, smart phone addiction, and NSSI, and social anxiety positively predicts the levels of smart phone addiction and NSSI. In addition, smart phone addictive behaviors exhibit a latent association with NSSI. Therefore, in the present study, we hypothesize that smart phone addiction and social anxiety may play a chain mediating role in peer aggression and NSSI.

### The moderating role of sex in this mediation model

**Fig. 1:**
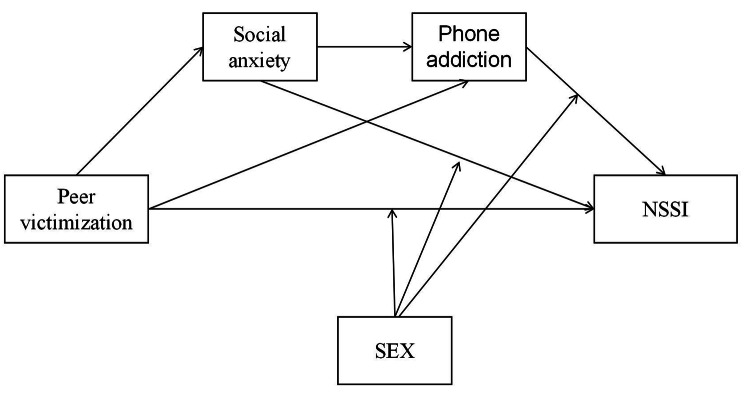
Model diagram for the chain mediating role of social anxiety and mobile phone addiction between peer victimization and NSSI and the moderating role of sex in peer victimization, social anxiety, and mobile phone addiction

Sex differences affect the prevalence of NSSI; however, the findings of previous studies in this regard have been inconsistent. Female students are also considered to be at risk of NSSI [2, 43, 44]. Conversely, a meta-analysis of self-injurious behavior in children and adolescents between 1989 and 2018 indicated that sex was not a significant moderator for explaining heterogeneity when suicidal and self-harming behaviors are prevalent [45]. This shows inconsistencies in the findings of studies on the incidence of self-harm between male and female students, which may be related to the differences in regions and samples, with some studies reporting that sex differences are more common in clinical samples than in community samples [46] and that sex differences are more common at younger ages [47]. Several studies have also investigated the relationship between peer victimization and NSSI as a function of sex. A cross-sectional survey of 578 adolescents in China showed that sex moderated the relationship between negative peer relationships and NSSI [48]. According to social role theory, this may be related to the fact that female students attach more importance to interpersonal relationships [49]. However, a 2022 meta-analysis could not verify the sex relationship between victimization and self-injury because fewer articles were included to increase the amount of sex difference between victimization and self-injury [50]. This study proposes an alternative hypothesis that sex plays a moderating role between peer victimization and NSSI. A survey on smart phone addiction found that boys tend to use gaming functions, whereas female students tend to use social functions, with female students having higher levels of addiction than male students(Yang et al., 2010). We speculate that sex may play a moderating role in cell phone addiction and NSSI. A study on adolescent social anxiety found that male students showed higher levels of social avoidance [51]; however, according to the above NSSI-mentioned interpersonal model, whether sex plays a moderating role remains to be clarified. Therefore, this study examined the moderating effect of sex between peer aggression and smart phone addiction. The integrated conceptual model of this study is presented in Fig. 1.

## Materials and methods

### Procedures and participants

All high schools in Zizhong County, a rural area, in central Sichuan were selected for this study, and all students enrolled in the school from April 13, 2022, to April 15, 2023 were surveyed. A total of 14,666 high school students were invited to participate in our anonymous survey, which was administered in classrooms. Informed consent was obtained from school administrators, students, and their parents prior to data collection. Classroom teachers or classroom leaders were first trained by trained leaders in the five high schools in Zizhong County to distribute the survey questionnaires. Students were informed that the collected data would be confidential and that they had the right to withdraw at any time. To ensure completeness and accuracy of data, students were given 1 h to answer the questionnaire questions centrally, all questionnaires were conducted in the Chinese language. To prevent any potential influence from teachers, alternative staff members were tasked with distributing and collecting the questionnaires. During the next 1 month, all participants were educated about emotion management and provided a free psychological hotline service. This study was approved by the Ethics Committee of Mianyang Third People’s Hospital-Sichuan Provincial Mental Health Center (Approval Document No: 2022 Annual Review;Ethics approval number:12510600451210007X.).

### Measures

#### Demographic characteristics

We used a self-administered questionnaire to collect sociodemographic information from the students, including sex, age, grade, class, and parents’ marital status (married, divorced, other), whether they were left behind (unable to live with both parents or either of them for more than six months due to work and other reasons).

#### Adolescent NSSI questionnaire

The NSSI scale [52] for adolescents was used to measure the self-injurious behaviors of secondary school students. Homogeneity reliability in a sample of Chinese adolescents was reported to be 0.85 [52]. It comprised two parallel paired parts: the number of self-injurious behaviors was assessed into four levels: 0, 1, 2–4, and ≥ 5, and the degree of physical injury was assessed into five levels: none, mild, moderate, severe, and very severe. This included 18 types of self-inflicted wounds such as intentionally scratching one’s skin with glass and knives and intentionally poking open wounds to prevent healing. The total score of the questionnaire was taken as the sum of the product of the number score and the grade score; as long as one of the scores was not zero, it was considered self-injurious behavior. A higher score indicated more serious self-injurious behavior. The value of Cronbach’s alpha and construct validity of the scale were 0.899 and 0.742, respectively [52].

#### Peer victimization questionnaire

The multidimensional peer victimization scale developed by Mynard and Joseph [53] and revised by Zhang [54] was used for measuring peer victimization levels. The internal consistency reliability of the physical aggression subscale in the Chinese adolescent sample reported in a previous study was 0.71, with the internal consistency reliability of the relationship aggression subscale being 0.85 [54]. It comprises two subscales of physical victimization and relational victimization, with 11 entries, and a 4-point Likert scale, with 0 indicating never and 3 indicating frequently. This included items such as other students threatening to beat me up in the last six months, other students deliberately ignoring me, etc. Higher scores indicated more severe peer victimization. The value of Cronbach’s alpha for this scale was 0.873, and the construct validity of the scale was 0.909.

#### Social anxiety questionnaire

The interaction anxiousness scale (IAS) questionnaire [55] uses a clinical empirical approach and specifically measures subjective feelings of anxiety about interpersonal interactions. Homogeneity reliability in a sample of Chinese adolescents was reported to be 0.87 [56]. It contains questions about everyday social situations that may provoke feelings of anxiety. For example, I feel nervous even in informal situations; I feel nervous when I must talk to teachers, etc. The IAS is a measure of the tendency to experience subjective social anxiety independently of behavior. The value of Cronbach’s alpha for this scale was 0.787, and the construct validity of the scale was 0.919.

#### Mobile phone addiction questionnaire

The mobile phone addiction index (MPAI) [55], developed at the Chinese University of Hong Kong, was used to diagnose mobile phone addiction in adolescents and college students. Homogeneity reliability reported previously in a sample of Chinese adolescents was 0.86 [57]. It included 17 questions, for example, you have been told you spend too much time on your phone; your friends and family have complained about your use of your phone, etc. The total score was in the range of 17–85, with higher scores indicating a higher tendency of mobile phone addiction. A higher score indicated a higher tendency for mobile phone addiction. For scoring, a 5-point Likert scale was used, where 1 meant never and 5 meant always. The value of Cronbach’s alpha for this scale was 0.892, and the construct validity of the scale was 0.896.

### Statistical analyses

All data in this study were analyzed using SPSS 26.0; continuous variables are expressed as mean ± standard deviation, whereas categorical variables are expressed as frequencies. To analyze the relationship between variables, Pearson correlation was used for data variables that conformed to a normal distribution, and Spearman correlation was used for variables that did not conform to a normal distribution. First, Model 6 in PROCESS macro was used to test the chain mediating effect. Bootstrap 95% confidence intervals (*CIs*) were used to examine whether the estimates of the chain mediating effect of the regression coefficients on 5000 samples were significant. The indirect effect was considered statistically significant if the 95% *CI* did not contain 0. Next, the moderating role of sex in the chain mediating effect was tested using Model 89, and bootstrap analysis was conducted using a heavy sample of 5000 95% *CIs* to analyze the significance of the mediating model with moderation. The Harman single-factor test was used for determining the common method bias [58]. Overall, there were 24 factors > 1 without rotation, and the first factor had an explanatory rate of 15.476%, which was less than the critical criterion of 40%, indicating that there was no significant common method bias in this study.

## Result

### Baseline information

Out of 14,666 questionnaires that were distributed, professionals proofread and selected 14,036 valid questionnaires after excluding non-response questionnaires (recovery rate: 95.7%). These valid questionnaires were collected from 6326 (45.1%) male students and 7710 (54.9%) female students, with participants’ age ranging from 15 to 18 (M = 16.40) years.

### Detection rates and sex differences

The detection rate of NSSI behaviors was 25.5%, with females having a higher detection rate (14.8%) than males (10.7%), and the difference was statistically significant (*P* < 0.001). Of these, 4.02% committed self-injury in only one way, 15.90% scored in the range 2–10, and 5.54% scored ≥ 10. The detection rate of peer victimization in this study was 65.1%, and the detection rate of physical victimization was 38.3%, with that for males being 21.8% and that for females being 16.6%, and the difference was statistically significant (*p* < 0.001). The detection rate of relationship victimization in this study was 56.6%, with that in males being 25.6% and that in females being 31.0%, indicating a non-significant sex difference (*p* = 0.63).

### Relevance analysis

Correlation analysis was performed on the variables of peer victimization, adolescent NSSI, social anxiety, and mobile phone addiction, and the results are presented in Table 1. A significant positive correlation was observed between peer victimization, NSSI, social anxiety, and mobile phone addiction (*P* < 0.001). Additionally, a significant positive correlation between sex (0 = male, 1 = female) and NSSI (*P* < 0.001).

**Table 1:** Spearman correlation analysis of variables

Variables	M	SD	1	2	3	4	5	6	7	8
Grade			1							
Marital status of parents			0.002	1						
Stay behind or not			0.031^***^	0.102^***^	1					
Sex			0.033^***^	0.002	0.040^***^	1				
Peer victimization	14.348	4.501	-0.084^***^	0.029^**^	0.025^**^	-0.026^***^	1			
Social anxiety	38.059	8.498	-0.056^***^	0.029^**^	0.075^***^	0.088^***^	0.180^***^	1		
Mobile phone addiction	43.010	12.320	-0.065^***^	0.059^***^	0.093^***^	0.013	0.337^***^	0.434^***^	1	
NSSI	1.820	6.051	− 0.071^***^	0.022^**^	0.032^***^	0.048^***^	0.326^***^	0.161^***^	0.244^***^	1

1 = Grade

2 = Marital status of parents

3 = Stay behind or not

4 = Sex

5 = Peer victimization

6 = Social anxiety

7 = mobile phone addiction

8 = NSSI

*n* = 14,036; *p<0.05, **p<0.01, ***p<0.001

### Chain mediated outcome test

Based on the results of the correlation analysis, after controlling for variables including grade level, whether the adolescent was left behind, and parental marital status, we considered peer victimization as a predictor variable, adolescent NSSI as an outcome variable, and social anxiety and mobile phone addiction as mediating variables. A chain mediated effects test was conducted using Model 6 in the SPSS PROCESS macro program. The results of the regression analysis are presented in Table 2. Peer victimization positively predicted adolescent NSSI (*β* = 0.363, *t* = 32.213, 95%, *CI* = [0.341, 0.385]), social anxiety (*β* = 0.328, *t* = 20.912, 95%, *CI* = [0.297, 0.359]), and mobile phone addiction (*β* = 0.723, *t* = 35.737, 95%, *CI* = [0.683, 0.763]). Social anxiety positively predicted mobile phone addiction (*β* = 0.551, *t* = 51.3523, 95%, *CI* = [0.530, 0.572]) and NSSI (*β* = 0.395, *t* = 6.338, 95%, *CI* = [0.0273, 0.052]). Mobile phone addiction positively predicted NSSI behavior (*β* = 0.062, *t* = 13.702, 95%, *CI* = [0.053, 0.071]). Mediated effects analysis revealed (Table 3) that social anxiety and mobile phone addiction significantly mediated the relation between peer victimization and NSSI, and social anxiety and mobile phone addiction serve as the chain mediators between peer victimization and NSSI. The total indirect effect value was 0.069, accounting for 19.01% of the total effect of peer victimization on NSSI.

**Table 2:** Regression analysis of the relationship of variables in the chain mediation model

Predictor variable	Outcome variable: social anxiety				Outcome variable: mobile phone addiction				Outcome variable: NSSI			
	β	SE	p	95% CI	β	SE	p	95% CI	β	SE	p	95% CI
Grade	-0.487	0.0919	<0.001	[-0.667-0.307]	-0.378	0.117	0.001	[-0.607 -1.489]	-0.292	0.062	<0.001	[-0.415 -0.170]
Marital status of parents	0.361	0.178	0.043	[0.012 0.710]	1.069	0.226	<0.001	[0.626 1.513]	0.0687	0.120	0.569	[-0.168 0.305]
Stay behind or not	1.238	0.146	<0.001	[0.952 1.524]	1.409	0.186	<0.001	[1.045 1.774]	0.124	0.099	0.201	[-0.701 0.319]
Peer victimization	0.328	0.157	<0.001	[0.297 0.359]	0.723	0.020	<0.001	[0.683 0.763]	0.363	0.113	<0.001	[0.341 0.385]
Social anxiety					0.551	0.107	<0.001	[0.530 0.572]	0.395	0.006	<0.001	[0.0273 0.052]
Mobile phone addiction									0.062	0.005	<0.001	[0.053 0.071]
Joint interpretative power	*R*^2^ = 0.040***				*R*^2^ = 0.263***				*R*^*2*^ = 0.131***			
Significance of the model	*F* = 144.222***				*F* = 998.894***				*F* = 351.515***			

**Table 3:** Bootstrap analysis of the intermediate effect test

	Indirect effect value	Boot SE	Boot LLCI	Boot UICI	Effect size
Total indirect effect	0.069	0.005	0.059	0.080	19.01%
Lnd1: Peer victimization→Social anxiety→NSSI	0.013	0.003	0.008	0.019	3.58%
Lnd2: Peer victimization→smart phone addiction→NSSI	0.045	0.004	0.036	0.053	12.40%
Lnd3: Peer victimization→Social anxiety→smart phone addiction→NSSI	0.011	0.001	0.009	0.014	3.03%

### Results of the moderated chained mediated effects test

After controlling for grade, whether left behind, and parental marital status, the measures were data-centered and tested for moderating effects using Model 89 in the SPSS PROCESS macro program. Sampling was repeated 5000 times to test the moderating role of sex in the chain mediated model (Fig. 1), and the results are presented in Table 4. After sex was given as input to the model, the product term of peer victimization and sex significantly affected NSSI (*β* = 0.114, *t* = 5.039, 95% CI = [0.070, 0.168]), and the product of mobile phone addiction and sex (*β* = 0.025, *t* = 2,710, 95% CI = [0.007, 0.042]) were significant predictors of NSSI. However, the product term of social anxiety and sex was not a significant predictor of NSSI (*β* = 0.004, *t* = 0.342, 95% CI = [− 0.203, 0.029]). Thus, sex can play a moderating role in the direct effect of peer victimization on adolescents’ NSSI behavior and can also moderate the predictive effect of smart smartphone addiction on NSSI behavior.

**Table 4:** Regression analysis of the relationship between variables in the chained mediation model with adjustment

Outcome variable	Predictor variable	β	SE	p	95% CI
Social anxiety	Peer victimization	0.328	0.157	<0.001	[0.297, 0.359]
	Stay behind or not	1.238	0.146	<0.001	[0.952, 1.524]
	Marital status of parents	0.361	0.178	0.043	[0.012, 0.710]
	Grade	-0.487	0.0919	<0.001	[-0.667, -0.307]
Smart phone addiction	Peer victimization	0.723	0.020	<0.001	[0.683, 0.763]
	Social anxiety	0.551	0.107	<0.001	[0.530, 0.572]
	Stay behind or not	1.409	0.186	<0.001	[1.045, 1.774]
	Marital status of parents	1.069	0.226	<0.001	[0.626, 1.513]
	Grade	-0.378	0.117	0.001	[-0.607, -1.489]
NSSI	Peer victimization	0.367	0.113	<0.001	[0.345, 0.389]
	Social anxiety	0.369	0.006	<0.001	[0.0245, 0.049]
	Mobile phone addiction	0.061	0.005	<0.001	[0.053, 0.070]
	Sex	0.603	0.096	<0.001	[0.450, 0.792]
	Ln1: Peer victimization × Sex	0.114	0.023	<0.001	[0.070, 0.168]
	Ln2: Social anxiety × Sex	0.004	0.013	0.732	[-0.203, 0.029]
	Ln3: mobile phone addiction × Sex	0.025	0.009	0.0067	[0.007, 0.042]
	Stay behind or not	0.105	0.099	0.291	[-0.089, 0.299]
	Marital status of parents	0.041	0.120	0.7362	[-0.195, 0.277]
	Grade	-0.305	0.062	<0.001	[-0.427, -0.183]

**Fig. 2:**
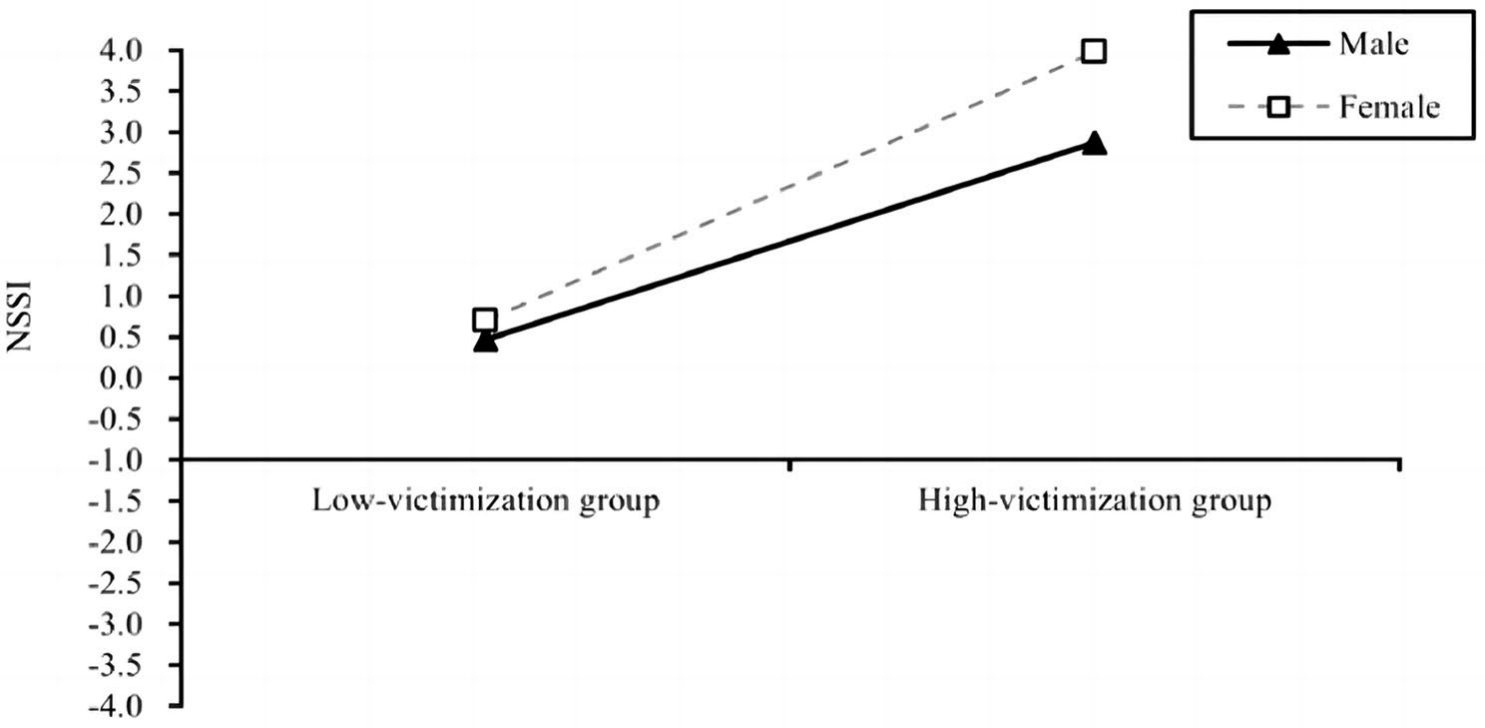
Model of the test for simple slopes showing the moderating role of sex in the association between peer victimization and NSSI

**Fig. 3:**
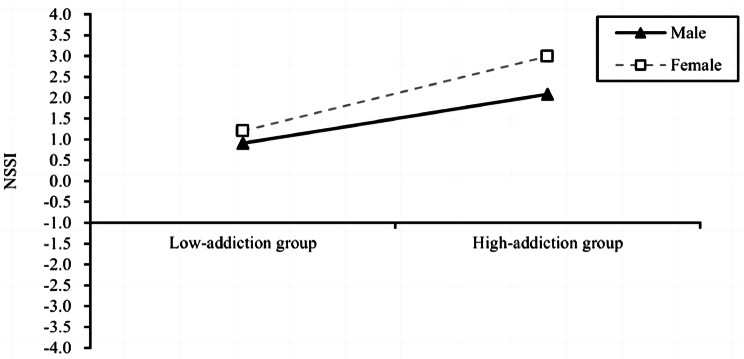
Model of the test for simple slopes showing the moderating role of sex in the association

Further simple slope analysis showed that the level of peer victimization in female positively predicted NSSI behavior (*β* = 0.418, *t* = 27.629, 95% CI = [0. 389, 0.448]), and the positive prediction effect was weaker in male (*β* = 0.305, *t* = 18.162, 95% CI = [0. 271, 0.337]). A simple slope analysis is shown in Fig. 2. Similarly, smart smartphone addiction levels among female students positively predicted their NSSI behavior (*β* = 0.072, *t* = 12.280, 95% CI = [0. 061, 0.084]), and smart smartphone addiction level among males also positively predicted NSSI behavior but with a smaller predictive effect (*β* = 0.048, *t* = 6.933, 95% CI = [0. 034, 0.061]). A simple slope analysis is shown in Fig. 3. Finally, we examined the Index of moderated mediation, and the results showed that in the model of chained mediated effects of social anxiety and smart phone addiction, the mediator index of moderation was 0.0044, (CI = [0.001, 0.009]).

Thus, the chain mediation model constructed in this paper was verified, indicating the moderating role of sex in peer victimization and smart smartphone addiction for predicting NSSI; moreover, social anxiety does not play a role in predicting NSSI.

## Discussion

In this study, we developed a moderated chain mediation model to explore the relationship between peer victimization and NSSI and the internal mechanisms of peer victimization and NSSI, as well as to identify priority populations. In addition, we report the detection rates and sex differences in peer victimization and NSSI, which helped us better understand the levels of peer victimization and NSSI in western China.

### Detection rates and sex differences in peer victimization and NSSI

In this study, we noticed that the detection rate of NSSI behaviors was 25.5%, which is similar to the results of a cross-sectional study in China (23.2%) [59] and another Chinese meta-analysis pooling the prevalence of 26 surveys, which showed an increase in NSSI prevalence from 2008 to 2016 with a pooled prevalence of (22.37%) [60]. In terms of sex, we found that females were more likely to engage in NSSI than males (*P* < 0.001), which is consistent with the results of a previous META and multiple cross-sectional survey analysis [43, 44, 61, 62]. In contrast, a previous study showed no significant difference in the prevalence of self-injury in the general population (*N* = 1442) sample [63]. Our finding supports the notion that the rate of detection of NSSI is higher in female students than in male students, which may be explained by the fact that the subjects of these students were adolescents studying in high schools. Previous studies have shown that the sex difference in NSSI is not a static process but may expand in adolescence and disappears in early adulthood [64]. The detection rate of peer victimization in this study was 65.1%, and the prevalence of different forms of peer aggression among European adolescents was reported to be 9.4– 33.0% [17]. A meta-analysis in China showed that the prevalence of peer victimization in mainland China was 2–66% [65]. and the detection rate of peer victimization in this study was at a higher level, While the high level of detection of peer victimization in this study is consistent with our conjecture that our sample originated from counties in western China, a less economically developed rural area with more left-behind children, which is consistent with previous findings, however, this inference needs more data to be verified. The detection rate of physical victimization was 38.3%, among these, the proportion of males was significantly larger than females, which is consistent with the findings of most previous studies, where the level of physical victimization was reported to be higher in males than in females [53, 66, 67]. The detection rate of relationship victimization in males and females showed non-significant sex difference, this may be because males focus more on physical aggression and may underestimate the level of relational victimization when they are subjected to physical versus relational aggression [67]; as a result, even if they are subjected to the same level of relational aggression, it is more likely that they will ignore it, compared to physical aggression.

### Social anxiety and the chain mediating role of cell phone addiction

Bourgeoning literature implied a positive correlation between social anxiety and high-risk behaviors [26]; [30, 31]. In the present study, among several indicators such as grade, marital status of parents, stay behind or not, sex, peer victimization, social anxiety, mobile phone addiction and NSSI, most significant positive correlation between peer victimization and NSSI was found, indicating that a high level of peer victimization increases the probability and frequency of self-injury, which is consistent with the findings of previous studies [20, 21]. As expected, peer victimization was positively correlated with NSSI. Here, our findings suggest that social anxiety plays a mediating role between peer victimization and NSSI. Nock indicated that individuals whose NSSI is influenced by interpersonal environments may engage in NSSI behaviors to avoid socialization [23]; in other words, these individuals may avoid socialization and engage in NSSI behaviors.

According to recent studies, there is a positive correlation between mobile phone addiction and NSSI among adolescents. Also, it was found that smartphone addiction could positively predict NSSI, particularly in pre-adolescence as compared to adolescents, which was related to low self-control and emotion dysregulation(Wang et al., 2022; Xu et al., 2023). Here, our data showed that mobile phone addiction mediates the relationship between peer victimization and NSSI behavior. First, we found that the level of peer aggression was significantly and positively associated with cell phone addiction. A longitudinal study showed that the level of peer victimization can predict the level of mobile phone addiction [39]. According to the Internet compensation theory, upon experiencing psychosocial problems, individuals may turn to the Internet or cell phones to escape the predicament they are in, which may be one of the reasons for the high level of cell phone addiction in peer-victimized individuals [68]. The results of this study showed that the higher the level of cell phone addiction, the higher the levels of NSSI. This is consistent with the findings of some studies, pointing out that cell phone addiction can lead to a range of problems such as anxiety, depression, and self-injury [69, 70]. The risk of self-injury induced by smart phone addiction may be related to the sleep problems caused by it. Sleep problems seriously affect adolescents’ mental health, and previous studies have indicated a significant positive correlation between sleep problems and NSSI [71], while smart phone use has been shown to significantly reduce sleep quality and decrease sleep length, among other issues. Our study also reports the establishment of a chain mediating effect in the same social anxiety and cell phone addiction in peer victimization and NSSI, suggesting that there is an association between these variables. Among these, mobile phone addiction showed the highest R2 of 0.263. The results of previous studies between social anxiety and cell phone addiction have been inconsistent, with some studies showing a strong positive correlation, whereas others reporting weak to no correlation between the two. This discrepancy in the findings may be related to differences in the study population; this study was conducted among high school students in Western China, and research suggests that Easterners exhibit a stronger sense of community and tend to experience more social anxiety, with Eastern culture involving communication through non-verbal language. Possibly, social anxiety and cell phone addiction are more closely linked in the aforementioned cultural context.

### The moderating role of sex in this mediation model

We finally investigated the moderating relationship of sex with the aforementioned issues and self-injury, indicating that sex moderated the relationship between peer victimization and NSSI, with female students’ peer victimization having a greater direct effect on their NSSI behavior. This phenomenon can be explained by the sociological theory, according to which female students tend to be more easily influenced by interpersonal relationships than male counterparts [49]. In other words, female students are more likely to experience adverse emotions when exposed to the same level of victimization. A previous study showed that female students exhibit lower emotional regulation than male students when experiencing negative peer relationships [72]. Another study found regional gray matter volume in the right dorsolateral prefrontal cortex in male students and in the left brainstem extending into anatomical clusters in the left hippocampus, left amygdala, and insular cortex in female students, which implies that male students may be more likely to withdraw from negative emotions [73]. The emotion regulation model of self-injury also states that negative emotions and the ability to regulate them play a crucial role in self-injury. In addition, the results suggest that the direct effect of mobile phone addiction levels on NSSI behavior is greater in female students than in male students. This may also be related to female’s poor ability to regulate negative emotions, as studies have found that cell phone addiction increases the risk of self-harm possibly because of the negative emotions it brings(Yang et al., 2010). This may be explained as differences in how mobile phone addiction manifests in male and female students, resulting in different addiction patterns. Males consider their mobile phones to be tools, whereas females use them mostly for social functions. Studies have also concluded that females are more dependent on smart mobile phones than males, which may explain the potential moderating role of sex in the aforementioned chain-mediated model [74, 75]. Female’s mobile phone addiction levels are more responsive to social problems than those of males. Another study also showed that the negative consequences of mobile phone addiction were more pronounced among females [76].

## Limitations and practical implications

This study has several limitations. First, the cross-sectional survey we used does not allow us to establish causal inferences about the observed associations, warranting longitudinal studies to validate our theoretical model in the future. Second, this study used a self-report approach that may have concealed self-injurious behavior, and in the future, multiple assessment methods, such as conversation method, observation method, and family report questionnaire, can be designed to make the theoretical model more reliable. Finally, relationship victimization and physical victimization in peer victimization were not viewed separately, and the theoretical model of this study counted the total victimization score, which can be refined in future experimental designs so that the intervention can be justified according to different types of victimization.

The present study has important practical implications. Although we did not involve the clinical population, the study findings provide insights for practice. First, the detection rate of peer victimization in western China is high; therefore, reducing a series of psychological problems brought about by peer victimization in the region is essential. This study is the first to include social anxiety and cell phone addiction in the model of peer victimization and NSSI, which provides new intervention methods to reduce the self-injury problems caused by peer victimization, such as group counseling and individual counseling to reduce social anxiety, or cell phone use management to reduce the length of cell phone use. We also established the relationship between social anxiety and cell phone addiction due to cultural differences and differences in communication styles [77, 78]. Furthermore, we analyzed the role of sex difference in peer victimization and NSSI and examined its moderating effect, which helped us better understand the relationship between these variables. Teachers and parents should focus more on adolescent female students who are peer victimized. More importantly, cognitive behavioral therapy should be provided to adolescents with peer problems with NSSI, which may reduce their levels of social anxiety, eventually alleviating their internalized psychological problems.

## Conclusion

In this cross-sectional study of 14,036 Chinese high school students, the detection rate of self-injury was on par with those reported in previous studies, and the detection rate of peer victimization was found to be higher. Peer victimization directly predicted NSSI and was indirectly predicted through social anxiety and mobile phone addiction. Further, according to simple slope analysis, peer victimization and mobile phone addiction levels among female students showed a less significant predictors of NSSI than among males.

## Data Availability

The datasets used and/or analyzed during the current study are available from the corresponding author upon reasonable request.
